# Visceral Adiposity, Pro-Inflammatory Signaling and Vasculopathy in Metabolically Unhealthy Non-Obesity Phenotype

**DOI:** 10.3390/diagnostics11010040

**Published:** 2020-12-29

**Authors:** Meng-Ting Tsou, Chun-Ho Yun, Jiun-Lu Lin, Kuo-Tzu Sung, Jui-Peng Tsai, Wen-Hung Huang, Chia-Yuan Liu, Charles Jia-Yin Hou, I.-Hsien Tsai, Cheng-Huang Su, Chung-Lieh Hung, Ta-Chuan Hung

**Affiliations:** 1 Department of Family Medicine, MacKay Memorial Hospital, Taipei 10449, Taiwan; mttsou@gmail.com; 2 Department of Nursing, Mackay Junior College of Medicine, Nursing and Management, New Taipei 25245, Taiwan; 3 Department of Medicine, Mackay Medical College, New Taipei 25245, Taiwan; med202657@gmail.com (C.-H.Y.); 8905012@gmail.com (K.-T.S.); 5505949.5949@mmh.org.tw (J.-P.T.); 5819.5819@mmh.org.tw (W.-H.H.); henry76@mmh.org.tw (C.-Y.L.); jiayin@mmh.org.tw (C.J.-Y.H.); chsu007@gmail.com (C.-H.S.); 4 Department of Radiology, MacKay Memorial Hospital, Taipei 10449, Taiwan; 5 Division of Endocrinology and Metabolism, Department of Internal Medicine, MacKay Memorial Hospital, Taipei 10449, Taiwan; jiunlulin@gmail.com; 6 Cardiovascular Division, Department of Internal Medicine, MacKay Memorial Hospital, Taipei 10449, Taiwan; 7 Division of Gastroenterology, Department of Internal Medicine, MacKay Memorial Hospital, Taipei 10449, Taiwan; 8 Nutritional Medicine Center, MacKay Memorial Hospital, Taipei 10449, Taiwan; a590214@mmh.org.tw; 9 Institute of Biomedical Sciences, Mackay Medical College, New Taipei 25245, Taiwan

**Keywords:** coronary artery calcification score (CACS), visceral adiposity, obese phenotype, metabolically unhealthy, hs-CRP

## Abstract

The debate regarding the actual cardiovascular burden in metabolically healthy obese or metabolically unhealthy non-obesity individuals is ongoing. Accumulating data have suggested a unique pathophysiological role of pro-inflammatory cytokines in mediating metabolic and cardiovascular disorders by dysregulated visceral adiposity. To compare the burden of visceral adiposity, the inflammatory marker high-sensitivity C-reactive protein (hs-CRP) and the prevalent atherosclerotic burden in metabolically healthy obese (MHO) or metabolically unhealthy (MU) populations, were compared to those of metabolically healthy non-obesity subjects (MHNO). Coronary artery calcification score (CACS) and visceral fat, including pericardial fat (PCF)/thoracic peri-aortic fat (TAT), were quantified in 2846 asymptomatic subjects using a CT dataset. A cross-sectional analysis comparing CACS, inflammatory marker hs-CRP, and visceral fat burden among four obesity phenotypes (MHNO, metabolically unhealthy non-obesity (MUNO), MHO, and metabolically unhealthy obese (MUO)) was performed. Both MUNO and MUO demonstrated significantly higher hs-CRP and greater CACS than MHNO/MHO (adjusted coefficient: 25.46, 95% confidence interval (CI): 5.29–45.63; 43.55, 95% CI: 23.38–63.73 for MUNO and MUO (MHNO as reference); both *p* < 0.05). Visceral fat (PCF/TAT) was an independent determinant of MU and was similarly higher in the MUNO/MHO groups than in the MHNO group, with the MUO group having the largest amount. PCF/TAT, obesity, and MU remained significantly associated with higher CACS even after adjustment, with larger PCF/TAT modified effects for MU and diabetes in CACS (both p_interaction_ < 0.05). MU tightly linked to excessive visceral adiposity was a strong and independent risk factor for coronary atherosclerosis even in lean individuals, which could be partially explained by its coalignment with pathological pro-inflammatory signaling.

## 1. Introduction

Obesity in terms of excessive adiposity is emerging as a major public health issue linked to metabolic abnormalities and increased cardiovascular events [1]. However, the associations between body mass index (BMI) and cardiovascular risks are not straightforward. Obesity without metabolic disturbance defined as metabolically healthy obesity (MHO) has been shown to progress to metabolic unhealthy obesity (MUO) with increased cardiovascular risks [2,3]. Conversely, metabolic abnormality alone might also be clinically important and has been proposed as an alternative obese phenotype known as metabolic obesity [4]. A subset of individuals with metabolically unhealthy non-obesity (MUNO) may have higher cardiovascular risks [2,5,6,7,8,9,10]. As both MHO and MUNO groups have shown potentially higher cardiovascular risks compared to those deemed to be metabolically healthy non-obesity (MHNO) subjects [7], data remain controversial and the exact underlying mechanisms are not clear [10,11,12].

Previous studies have demonstrated that central fat [13], especially dis-regulated visceral adiposity, was a key pathophysiological factor that determined metabolic abnormality and unfavorable cardiovascular outcomes [14]. However, central obesity assessed by waist circumference (WC) may not accurately reflect the true burden of visceral adiposity [15]. Recently, multi-detector computed tomography (MDCT) has rendered objective quantification of visceral fat (pericardial fat (PCF) and thoracic peri-aortic adipose tissue (TAT)) feasible [16,17], and this value has been shown to play an independent role in the pathogenesis of atherosclerosis [18]. Recently, racially diverse associations among obesity phenotypes and cardiovascular risks have been proposed, for example, Asian populations may present with higher prevalent metabolic and cardiovascular diseases with lower BMI compared to that of white populations [3,19]. Coronary artery calcification (CAC) as a clinical surrogate for coronary artery disease (CAD) and has been recognized as a robust predictor of vasculopathy and atherosclerosis tightly linked to outcomes [3,20], though comprehensive data comparing visceral fat and CAC burden between MUNO and MHO remains largely unexplored.

The aim of this study was to investigate the associations of different obesity phenotypes according to clinical metabolic and BMI strata with visceral fat burden and prevalent coronary atherosclerosis. We further assessed their roles in pro-inflammatory signaling in a cross-sectional fashion.

## 2. Materials and Methods

### 2.1. Study Population

From 2005 to 2009, MDCT was performed to assess the coronary calcium levels of 3116 study participants (age: 48.37 ± 8.32 years; 27.62% females) who participated in a cardiovascular health survey program at a tertiary medical center in Taipei, Taiwan. Subjects were excluded if they had any of the following issues: (1) missing baseline BMI (weight in kilograms divided by the square of the height in meters) or missing information about the metabolic components; (2) acute coronary syndrome; (3) acute decompensated congestive heart failure; (4) underweight (BMI < 18.5); and (5) current use of lipid-lowering drugs (i.e., statins or fibrates). After exclusion, a total of 2846 subjects (age: 49.5 ± 8.3 years; 27.6% females) were included in the present analysis. The study protocol was evaluated and approved by the Human Research Ethics Committee of Mackay Memorial Hospital (project research number 18MMHIS137, 15 Oct 2018). As a retrospective study design, informed consent was waived from our institutional board review. All study participants were de-identified during data analysis.

A detailed physical examination and a thorough review of the baseline characteristics and medical history (including smoking and physical activity status) were performed using structured questionnaires. A history of cardiovascular disease (CVD) was defined as previous myocardial infarction, CAD with coronary intervention, stroke, prior hospitalization for congestive heart failure, and known peripheral arterial disease. A history of hypertension (HTN) was defined as systolic blood pressure higher than 140 mmHg, diastolic blood pressure higher than 90 mmHg, or a previous diagnosis of HTN with current medications. A history of diabetes mellitus (DM) was defined as a fasting glucose level more than 126 mg/dL or the current use of any diabetic medication for treating previously diagnosed DM. Part of our data has been published elsewhere before [21].

### 2.2. Baseline Anthropometric Measurements

All baseline characteristics and anthropometric measurements, including age, body height, body weight (BW), BMI, waist circumference (WC), and buttock circumference, were collected. Height was measured to the nearest 0.01 cm by using a standard stadiometer. Weight was measured in light clothes to the nearest 0.01 kg by using a set of standard calibrated electronic scales. The WC and hip circumference (HC) were measured using a constant-tension tape. WC was measured at the midpoint between the lowest rib and the upper point of the iliac crest and at the end of normal expiration. HC was measured at the maximum protrusion of the buttocks. Standardized sphygmomanometer cuff-defined resting blood pressure values were measured while resting. Anthropometric measurements such as height, weight, WC, HC, and blood pressure were examined and recorded by trained nurses who were blinded to the patient’s information in a laboratory center.

### 2.3. Classification According to the Metabolic and Weight Status

Non-obesity (NO) was defined as BMI (kg/m^2^) < 27 (*n* = 2216; 77.86%) with obesity defined as BMI ≥ 27 (*n* = 630; 22.14%) according to the Asia-Pacific criteria [22]. Metabolic score was determined using the National Cholesterol Education Program-Adult Treatment Panel III classification with Taiwan-specific cutoffs for abdominal obesity [23]. Those who fulfilled metabolic syndrome criteria with at least three of the following five components were defined as metabolically unhealthy (MU): (1) WC ≥ 90 cm for men and > 85 cm for women; (2) high-density lipoprotein cholesterol (HDL-C) < 40 mg/dL for men and < 50 mg/dL for women; (3) triglyceride (TG) levels ≥150 mg/dL; (4) blood pressure ≥ 130/85 mmHg or treatment for HTN; and (5) fasting blood glucose (FBS) ≥ 100 mg/dL or treatment for type 2 DM [23]. Subjects were categorized into four groups according to presence of MU and non-obesity/obesity strata as: (1) MHNO; (2) MUNO; (3) MHO; and (4) MUO groups. The Framingham risk score (FRS) defines 10-year CVD risks with several criteria of MetS, such as total cholesterol, systolic blood pressure, diastolic blood pressure, and sex (for age) [24].

### 2.4. Laboratory Data Acquisition and Analysis

A Hitachi 7170 Automatic Analyzer (Hitachi Corp., Hitachinaka Ibaraki, Japan) was used to measure the levels of fasting glucose (hexokinase method), total cholesterol, TG, and uric acid (UA). Lipid profiles, including low-density and high-density lipoprotein cholesterol, were measured by homogenous enzymatic colorimetric assay. High-sensitivity C-reactive protein (hs-CRP) levels were determined using a highly sensitive latex particle-enhanced immunoassay analyzer (Elecsys 2010 analyzer: Roche, Mannheim, Germany) [18].

### 2.5. MDCT Scanning Protocol

Scanning was performed using a 16-slice MDCT scanner (Sensation 16; Siemens Medical Solutions, Forchheim, Germany) with 16- × 0.75-mm collimation, rotation time of 420 ms, and tube voltage of 120 kV. During one breath-hold, images were acquired from above the level of tracheal bifurcation to below the base of the heart by using prospective ECG-triggering with the center of the acquisition at 70% of the R–R interval. Using the raw data, the images were reconstructed with standard kernels in 3-mm-thick axial non-overlapping slices and a 25 cm field of view [18].

### 2.6. Coronary Artery Calcium (CAC) Measurements

We measured the calcification of all coronary arteries by using a dedicated offline workstation (Aquarius 3D Workstation; TeraRecon, San Mateo, CA, USA). A coronary calcified lesion was defined as an area with a density >130 HU and covering at least six pixels. Existence of clinically significant atherosclerosis was defined by any presence of CAC; the burden of atherosclerosis, defined by CAC score (CACS), was semi-quantitatively assessed using the Agatston score method by multiplying each lesion (area) by a weighted CT attenuation score of the lesion [18].

### 2.7. Measurements of PCF and TAT

**Figure 1:**
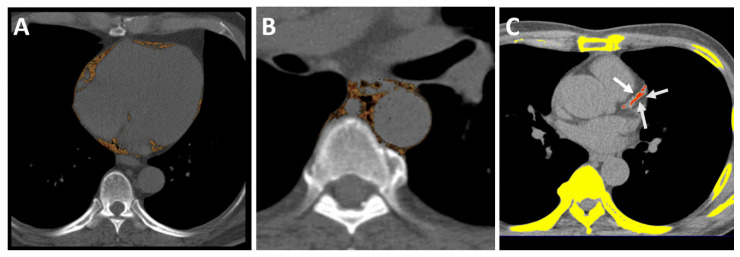
Multidetector computed tomography (MDCT) demonstrated visceral adiposity measures, including pericardial and thoracic peri-aortic fat tissue, and quantification of CACS. The pericardial fat was defined as the fat between the heart and the pericardium, as shown in axial view (**A**). (**A**) Pericardial adipose tissue. (**B**) Thoracic peri-aortic adipose tissue. (**C**) Semi-automatic quantification of CACS burden using Agatston scoring. *Orange color regions indicate visceral fat tissue. White arrows indicate coronary calcification lesions.

The visceral adipose tissues of PCF and TAT were quantified using MDCT using a dedicated workstation (Aquarius 3D Workstation). The semi-automatic segmentation technique was developed for the quantification of fat volumes. We traced the region of interest manually and defined the fat tissue as pixels within a window of −195 to −45 HU and a window center of −120 HU. PCF was defined as the volume-based burden of total adipose tissue located within the pericardial sac (Figure 1A). The TAT tissue was defined as the total adipose tissue volume surrounding the thoracic aorta (as peri-aortic fat), which extends 67.5 mm from the level of bifurcation of the pulmonary arteries (Figure 1B) with cranial-caudal coverage of the thoracic aorta [18]. We further indexed PCF and TAT measurements according to body surface area (PCFi and TATi, respectively) in the current study to perform comparisons of the relative composition of visceral adiposity in the same body size. 

### 2.8. Reproducibility for MDCT-Derived Visceral Adiposity

The reproducibility of PCF and TAT was assessed by repeated measurements of 40 randomized cases with the initial results and clinical data blinded between readers, and those results were published before [18]. The intra-observer and inter-observer coefficient of variation for PCF were 4.27%, 4.87% and for TAT were 6.58%, 6.81%, respectively.

### 2.9. Statistical Analysis

The baseline clinical, demographic, anthropometric, and laboratory data are presented as mean ± standard deviation for continuous variables; numbers and percentages are presented for categorical variables. Comparisons among the groups were calculated using a one-way analysis of variance for continuous variables; Pearson’s chi-square test was used to calculate categorical variables. Relationships among the four groups and the presence of CAC were estimated as an odds ratio (OR) with a 95% confidence interval (CI). Relationships among the four groups and burden of CAC (according to the CACS), PCF, and TAT were estimated as the regression coefficient. In the initial univariate analysis, a threshold of *p* < 0.20 was used to identify the confounding variables for inclusion in the final multivariate model. Multivariate linear regression was used to examine the independent associations between four obesity phenotypes with burden of atherosclerosis (CACS as continuous variable), visceral adiposity (PCF/TAT) and hs-CRP level, with multivariate logistic regression used to determine the independent associations between four obesity phenotypes and the presence of CAC (yes/no as a binary variable). The Framingham score was used as a clinical confounding covariate in multivariate models. As the presence of diabetes may not be deemed to be metabolically healthy clinically even without meeting criteria for MU, we further conducted sensitivity analysis to see whether these associations remained unchanged by excluding subjects with known DM or CVD. 

We performed all analyses using SPSS (IBM Corp., Armonk, NY, USA) for Windows. We considered two-sided *p* < 0.05 to be statistically significant.

## 3. Results

### 3.1. Baseline Characteristics of the Subjects

Among 2846 study participants who had complete baseline information and CACS available for the final analysis, MU was prevalent in 826 subjects (29.0%) (Table 1). The distribution of the four obesity phenotypes was: MHNO (63.0%), MUNO (14.9%), MHO (8.0%), and MUO (14.1%). Study participants in the MUNO group were oldest, with those in the MHO group being the youngest. Those in the MHO and MUO groups were more likely to be male. Metabolic abnormalities were most pronounced in MUO. MUNO and MUO groups had similarly high FRS, 10-year CVD risks, CACS, and comparable prevalent systemic and cardiovascular diseases when compared to those of the MHNO and MHO groups (Table 1). PCF and TAT were highest in MUO and were similarly higher in the MUNO and MHO groups compared to those of the MHNO group, even after multivariate adjustment (Supplemental Table S1). The MUNO and MUO groups had higher hs-CRP levels than those of the MHNO and MHO groups (*p* < 0.001). 

### 3.2. Associations between Four Obesity Phenotypes, Metabolic Abnormality, and CAC Burden

**Figure 2:**
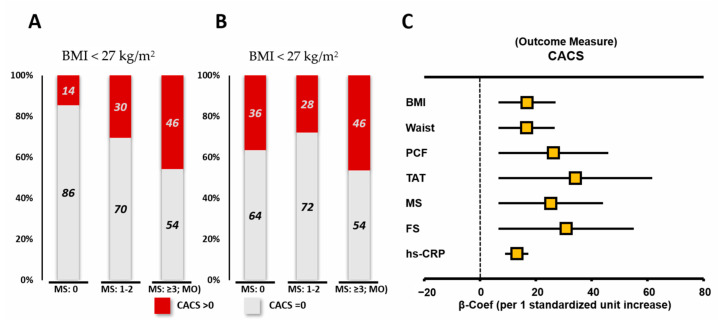
Prevalence of coronary artery calcification (CACS > 0, red) according to metabolic scoring and metabolic unhealthy (MU) according to the body mass index (BMI) strata (**A**,**B**). Associations of atherosclerotic burden (CACS) with per one standardized unit increase of anthropometrics, visceral adiposity, and cardiovascular risk scoring (**C**). FS: Framingham score, PCF: pericardial fat, TAT: thoracic peri-aortic fat, MS: metabolic score.

Study participants with MU (MUNO/MUO) had markedly higher CACS severity than that of other groups without MU (MHNO/MHO) (Table 2). Those in the MUO group had the highest CACS severity, and were more likely to have CAC, particularly for those younger (<55 years), males, and those without DM and HTN, when compared to members of the other three groups (Supplemental Table S2). Sensitivity analysis confirmed these findings by excluding known diabetes and CVD (adjusted estimates for CACS: 26.9, 48.2, 37.0, 60.1 across the four sequential groups as in Table 1, *p* < 0.001). Instead, females in the MUNO/MHO groups showed higher CACS severity (Table 2). Despite the markedly low prevalent CAC of the non-obese group without a metabolic score (MS) (as 0), surprisingly, CAC was equally prevalent in the obese and non-obese groups presenting higher metabolic scores (MSs, i.e., ≥3, as MU) (Figure 2A,B). A graded increase of prevalent CAC was observed with increasing MS in both NO and obesity populations (both *p* for trend: <0.05, Supplemental Figure S1A). Likewise, subjects with lower PCF/TAT had a lower yet similarly graded increased prevalence of coronary calcification with increasing metabolic scores compared to those of the higher PCF/TAT groups (Supplemental Figure S1B). Figure 2C demonstrates the standardized coefficient values of anthropometric assessment (BMI and WC), visceral adiposity measurements (PCF and TAT), and cardiovascular risk scores (MS and Framingham score (FRS)) in relation to CACS burden. The results showed the order of the influencing weighs for CACS as follows: TAT > FS > PCF > MS > BMI/waist.

### 3.3. Associations between Four Obesity Phenotypes, Metabolic Abnormality, and hs-CRP Level

Both MUNOW and MUO signaled higher systemic inflammation in terms of higher hs-CRP compared to MHNO/MHO after full adjustment, with MUO demonstrating higher systemic inflammation across all subgroup analyses; in contrast, MHO showed markedly systemic inflammation in younger and female subgroups (Table 2). Compared to MHNO, those in MUNO demonstrated higher hs-CRP in older, male, non-diabetic and non-CVD study participants (Table 2, all adjusted *p* < 0,05). By multivariate adjustment, obesity, presence of MU, and higher TAT (adjusted coefficient: 0.01 (95% CI: 0.009, 0.02), 0.10 (95% CI: 0.05, 0.15), both *p* < 0.001, and 0.01 (95% CI: 0.003, 0.01), *p* = 0.002) were all independently associated with higher hs-CRP, with only TAT associated with higher hs-CRP in those with CVD (adjusted interaction coefficient: 0.20 (95% CI: 0.01, 0.39), p_interaction_: 0.036). 

### 3.4. Associations between Adiposity, Metabolic Abnormality, and CAC Burden

**Figure 3:**
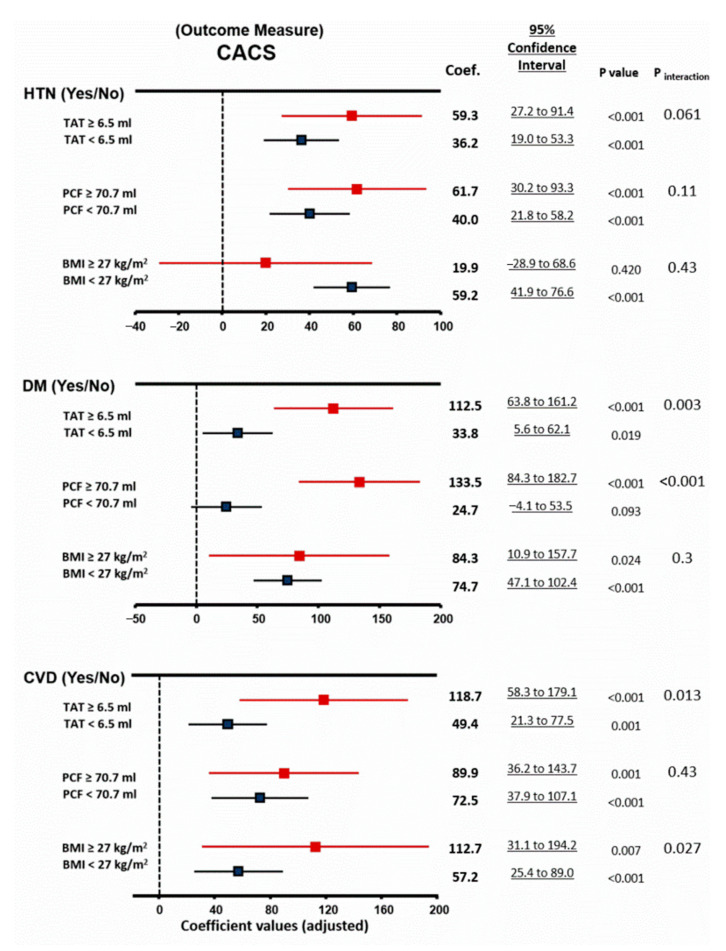
Associations of coronary artery calcification scores (CACS) with physical obesity from Table 3.

Presence of MU and obesity groups were consistently associated with significantly greater CACS even after adjustment for all subjects, regardless of age or sex strata and between sub groups (Table 2 and Table 3, Supplemental Table S3). Overall, compared to PCF, TAT demonstrated more significant associations with CACS, which was more pronounced in subjects presenting as MU (all P_interaction_: < 0.05, Table 3). Presence of metabolic unhealthy (MU) modified the effects between greater PCF/TAT, but not BMI, and CACS burden (Table 3 and Figure 3) (both p_interaction_ < 0.05). Larger PCF and TAT also strongly accentuated the impacts of DM on CACS burden (Figure 3), with larger TAT and obesity associated with higher CACS for individuals with prevalent CVD (all p_interaction_ < 0.05). These findings showed that assessing PCF and TAT may provide additional values beyond BMI for atherosclerotic burden (CACS) and seemed to be more pronounced in those clinically presenting as MU.

## 4. Discussion

To the best of our knowledge, this is the first study to explore atherosclerotic burden in terms of CAC severity relating to visceral adiposity and inflammation across diverse clinical obesity phenotypes. The main and novel findings were as follows: MUNO may exhibit a similarly greater coronary atherosclerotic burden (measured via CACS) and higher inflammatory marker (hs-CRP) as MUO when compared to MHNO/MHO even after correcting for age and baseline characteristics, with MUO individuals demonstrating the highest CACS; despite the lower BMI, subjects in the MUNO group may have a visceral fat burden as high as those in MHO and MUO groups when compared to those in the MHNO group; MU as an alternative clinical obesity phenotype may confer the same risk for coronary atherosclerosis in both normal weight and obese individuals; and greater visceral adiposity, especially TAT, may further accentuate the negative impacts of metabolic abnormality or diabetes on CACS severity. 

Consistent with previous studies, both the MHO and MUO groups in the present work had abundant visceral fat accumulation [25,26]. We further observed that visceral fat burden was comparable among MUNO, MHO, and MUO groups, and was markedly higher when compared to that of the MHNO group, even after full adjustment. Notably, the relative proportions of the four obesity phenotypes in this study were comparable with previous study in white populations [9], indicating our defined obesity subgroups were not racially deviated. Despite greater central obesity and body weight in both MUO and MHO groups, greater atherosclerotic burden in terms of higher CACS was mainly observed in individuals manifesting as metabolic unhealthy (MUONO and MUO) and with enhanced systemic inflammation, an alternative obesity phenotype known as metabolic unhealthy (MU). These findings may partially explain MHO as a relatively benign condition for cardiovascular diseases, as the MHO group in present study had CACS higher than that of the MHNO group but lower than that of the MUNO and MUO groups [5,27,28]. Likewise, presence of MU seemed to be a major risk for CACS [18], even after adjusting for the confounding variables. These findings were also in agreement with those of previous studies showing that MUO and MUNO groups may be predisposed to CVD [2,29,30,31], thus highlighting the presence of metabolic disorders (i.e., MetS) from circulating oxidative apolipoproteins as the main atherosclerotic risk factor initiating foam cell formation and plaque progression [32,33,34].

Central obesity in terms of higher waist-to-hip ratio or WC is also a well-documented anthropometric factor associated with diabetes and more severe CVD [35,36]. Compared to the MHO and MUO groups, individuals in MUNO group exhibited smaller WC yet comparable visceral adiposity. These findings indicated that central obesity by anthropometric measure may not be a useful indicator, nor was it a reliable clinical surrogate, for identifying individuals at higher atherosclerotic risk. On the contrary, excessive visceral adiposity can signal numerous activated circulatory pro-inflammatory adipocytokines, decreased eNOS activity, unfavorable lipid profiling, and disrupted integrity of vascular endothelium leading to accelerated “vasculopathy” [37,38]. This was partially evidenced by similarly redundant visceral fat between the MUNO and MHO groups, and slightly higher hs-CRP levels in both the MUNO and MUO groups [23]. Notably, presence of MU seemed to modify the associations between PCF/TAT and CACS (strong risk factors for CVD) but not BMI, supporting the concept that dysregulated visceral adiposity rather than larger BMI may aggravate atherosclerotic pathology (Table 3). Therefore, assessment of PCF and TAT will likely be of additive clinical value in identifying subjects at higher atherosclerotic risk.

It has been proposed that Asian populations are more prone to metabolic disorders, diabetes, and heightened inflammatory status and are more susceptible to CVD than are Western populations with a lean body size presenting with relatively abundant central or visceral obesity, known as the “thin-fat phenotype.” [39,40,41]. Compared to the MHNO group, the MUNO and MUO groups had relatively higher hs-CRP levels, which may promote endothelial dysfunction and coronary atherosclerosis (the pathogenesis of both glucose intolerance and atherosclerosis) [42]. Several putative mechanisms can explain the finding that MUNO, MHO, and MUO are associated with adverse CACS profiles from redundant visceral adipose tissue depots. Indeed, our results, together with those of previous studies, suggested that central obesity or body fat content might be clinically more important than BMI. Our findings also indicated that visceral fat amount assessed by MDCT could play a central role in this clinical implication [43]. Overall, these findings emphasized metabolic abnormality and excessive visceral fat irrespective of obesity as the driving pathological determinant of atherosclerosis in Asian populations.

Compared to previous studies [31,37], our study had some important strengths. First, all subjects underwent non-invasive determination of CACS, PCF, and TAT for the exact evaluation of CAD burden and visceral adipose tissue accumulation. We included a large-scale asymptomatic population (*n* = 2846) in our study; the sample size was probably large enough to explore the potential associations between the study variables. Second, we assessed the CACS severity and the presence of CACS. PCF severity and TAT severity were evaluated. Third, data of the total population and the presence of obesity (defined by BMI), DM, HTN, and CVD were evaluated. 

## 5. Limitations

There were some potential limitations to our study. First, according to current research, the prevalence of MUNO and MHO phenotypes depends on the diagnostic criteria [5]. Our results might have varied according to the definition of obesity. Second, the percentage of body fat in Asian populations is usually higher than that in European populations, and there is currently no consensus regarding the optimal cutoff for BMI to define “overweight” and “obesity” in Asian populations [44]. The risk of DM, HTN, and hyperlipidemia with a relatively low BMI in the Asian population is increasing [44]. Therefore, the BMI cutoff points for obesity for the Asian populations will be lower than that for the European population. Moreover, there was no distinction between “overweight” and “obese” groups in our study. Third, cross-sectional studies are unlikely to infer causality from the associations described. Ultimately, large-scale, prospective trials may be required. Fourth, whether these results can be extended to races other than Taiwanese and other East Asians is unknown. Finally, we also need to acknowledge the lack of other supporting biomarkers (such as IL-1 or IL-6) in our current study to further strengthen the role of pro-inflammatory cytokines in mediating atherosclerotic pathophysiology underlying excessive visceral adiposity [45]. 

## 6. Conclusions

MUNO and MU were associated with the prevalence and severity of CAC. MHO and obesity were associated with the severity of PCF and TAT after adjustment for variables. In particular, these associations were generally consistent among subjects without DM, HTN, and CVD. Our study further showed that excessive visceral adiposity likely plays a role in the pathogenesis of atherosclerosis through pro-inflammatory signaling, especially for those subjects manifesting metabolically *unhealthy* phenotypes. Additionally, MUNO and MHO individuals should be more carefully examined when assessing risk factors for CAC or atherosclerosis, irrespective of DM, HTN, or CVD.

## Figures and Tables

**Table 1:** Baseline characteristics of the study population.

	MHNO	MUNO	MHO	MUO	*p* Value
	(*n* = 1793)	(*n* = 423)	(*n* = 227)	(*n* = 403)	
Age, years	48.27 ± 9.14	53.26 ± 9.45 *	47.77 ± 9.83 ^†^	50.51 ± 9.69 *^,†,#^	<0.001
Male, n (%)	1231 (68.66)	312 (73.76)	176 (77.53)	341 (84.62)	<0.001
BH (cm)	165.74 ± 8.03	166.58 ± 8.47	166.24 ± 7.92	167.77 ± 7.89	<0.001
BW (kg)	63.22 ± 9.23	69.03 ± 8.88 *	80.04 ± 9.76 *^,†^	84.76 ± 11.10 *^,†,#^	<0.001
BMI (kg/m^2^)	22.92 ± 2.23	24.78 ± 1.58 *	28.88 ± 1.93 *^,†^	30.05 ± 2.84 *^,†,#^	<0.001
WC (cm)	79.45 ± 8.32	87.11 ± 7.42 *	91.20 ± 12.95 *^,†^	97.23 ± 9.29 *^,†,#^	<0.001
WC/HC ratio	0.86 ± 0.07	0.92 ± 0.05 *	0.91 ± 0.06 *	0.94 ± 0.05 *^,†,#^	<0.001
SBP (mmHg)	118.25 ± 15.08	131.57 ± 16.95 *	123.66 ± 16.71 *^,†^	133.77 ± 15.95 *^,#^	<0.001
DBP (mmHg)	73.40 ± 10.00	80.88 ± 10.90 *	77.74 ± 10.09 *^,†^	83.27 ± 10.09 *^,†,#^	<0.001
FBG (mmol/L)	96.38 ± 14.45	113.26 ± 29.54 *	96.11 ± 8.62 ^†^	117.02 ± 33.71 *^,#^	<0.001
UA (mg/dL)	5.75 ± 1.37	6.33 ± 1.47 *	6.34 ± 1.43 *	6.78 ± 1.39 *^,†,#^	<0.001
TC (mg/dL)	200.07 ± 36.29	210.05 ± 36.34 *	199.50 ± 33.30 ^†^	203.72 ± 38.27	<0.001
TG (mg/dL)	113.13 ± 106.97	208.64 ± 109.57 *	122.66 ± 55.73 ^†^	212.11 ± 136.68 *^,#^	<0.001
HDL (mg/dL)	56.58 ± 14.07	44.89 ± 11.00 *	50.91 ± 10.99 *^,†^	42.41 ± 9.82 *^,#^	0.007
LDL (mg/dL)	129.07 ± 31.46	134.79 ± 33.40 *	131.55 ± 30.64	131.82 ± 33.69	<0.001
Diabetes, n (%)	41 (2.29)	61 (14.42)	2 (0.88)	59 (14.64)	<0.001
Hyperlipidemia, n (%)	47 (2.62)	51 (12.06)	6 (2.64)	47 (11.66)	<0.001
CVD, n (%)	40 (2.23)	37 (8.75)	7 (3.08)	41 (10.17)	<0.001
Stroke, n (%)	5 (0.28)	4 (0.95)	1 (0.44)	3 (0.74)	0.240
Smoking, n (%)	185 (10.32)	66 (15.60)	30 (13.22)	62 (15.38)	0.002
Alcohol, n (%)	89 (4.96)	24 (5.67)	19 (8.37)	37 (9.18)	0.004
Exercise, n (%)	267 (14.89)	65 (15.37)	30 (13.22)	65 (16.13)	0.794
Framingham score	1.98 ± 4.39	6.39 ± 3.38 *	3.04 ± 3.38 *^,†^	5.74 ± 3.26 *^,#^	<0.001
Framingham 10-yr CVD risk (%)	5.21 ± 4.03	10.93 ± 7.43 *	5.89 ± 4.85 ^†^	10.40 ± 7.79 *^,#^	<0.001
Framingham 10-yr CVD ≧ 10%, n (%)	224 (12.49)	213 (50.35)	32 (14.10)	172 (42.68)	<0.001
hs-CRP (mg/dL)	0.16 ± 0.35	0.26 ± 0.64 *	0.24 ± 0.33	0.32 ± 0.42 *	<0.001
PCF	65.97 ± 25.44	84.13 ± 28.68 *	89.36 ± 29.29 *	101.18 ± 35.26 *^,†,#^	<0.001
PCFi	35.9 ± 13.1	44.0 ± 15.4 *	43.4 ± 14.4 *	47.6 ± 16.6 *^,†,#^	<0.001
TAT	5.73 ± 2.94	8.40 ± 3.58 *	8.62 ± 3.69 *	11.14 ± 4.95 *^,†,#^	<0.001
TATi	3.08 ± 1.49	4.35 ± 1.78 *	4.18 ± 1.77 *	5.22 ± 2.24 *^,†,#^	<0.001
CACS	22.25 ± 109.55	72.29 ± 231.59 *	44.94 ± 193.84	86.73 ± 310.35 *^,#^	<0.001
CACS, n (%)	440 (24.54)	193 (45.63)	64 (28.19)	186 (46.15)	<0.001

MHNO = metabolically healthy non-obesity group, MUNO = metabolically unhealthy non-obesity group, MHO = metabolically healthy obesity group, MUO = metabolically unhealthy obesity group, *n* = number, BH = body height, BW = body weight, BMI = body mass index, WC = waist circumference, WC/HC ratio = waist circumference/hip circumference ratio, SBP = systolic blood pressure, DBP = diastolic blood pressure, FBG = fasting blood glucose, UA = uric acid, TC = total cholesterol, TG = triglyceride, HDL-C = high density lipoprotein cholesterol, LDL-C = low density lipoprotein cholesterol, CVD = cardiovascular disease, hs-CRP = high-sensitivity C-reactive protein, PCF = pericardial fat, PCFi = indexed pericardial fat, TAT = thoracic peri-aortic adipose tissue, TATi = indexed thoracic peri-aortic adipose tissue, CACS = coronary artery calcification score. During the ANOVA analysis, values labeled with different superscripts in a row indicate significant differences between groups based on Scheffe’s multiple comparison test: * *p* < 0.05 vs. MHNO; † *p* < 0.05 vs. MUNO; # *p* < 0.05 vs. MHO.

**Table 2:** Associations between four obesity phenotypes, coronary artery calcification (CAC) burden (according to the CACS), and hs-CRP level.

	MHNO (*n* = 1793)	MUNO (*n* = 423)	MHO (*n* = 227)	MUO (*n* = 403)
	β (95% CI)	β (95% CI)	β (95% CI)	β (95% CI)
**CACS**				
Unadjusted	(reference)	50.03 (31.03, 69.04) ***	22.69 (−2.08, 47.45)	64.48 (45.10, 83.86) ***
Model1 †	(reference)	26.26 (7.45, 45.08) **	22.45 (−1.65, 46.56)	50.01 (30.95, 69.06) ***
Model2 ‡	(reference)	25.46 (5.29, 45.63) *	16.78 (−7.86, 41.41)	43.55 (23.38, 63.73) ***
Subgroup effects				
Age, years ‡				
<55	(reference)	9.93 (−6.45, 26.32)	2.92 (−15.17, 21.02)	33.67 (18.05, 49.30) ***
≥55	(reference)	60.09 (6.34, 113.83) *	80.27 (0.83, 159.72) *	87.64 (29.66, 145.62) **
Sex ‡				
Female	(reference)	31.85 (5.93, 57.77) *	54.86 (21.79, 87.94) **	45.62 (13.48, 77.76) **
Male	(reference)	15.22 (−10.69, 41.12)	7.03 (−23.90, 37.95)	33.22 (8.65, 57.78) **
Diabetes ‡				
No	(reference)	9.60 (−8.17, 27.38)	17.90 (−2.81, 38.61)	27.87 (10.01, 45.73) **
Yes	(reference)	159.88 (−19.92, 339.69)	−42.19 (−655.29, 570.92)	165.35 (−11.14, 341.84)
Hypertension ‡				
No	(reference)	3.97 (−15.85, 23.79)	20.80 (−1.00, 42.60)	31.76 (11.39, 52.13) **
Yes	(reference)	42.26 (−22.53, 107.05)	−5.28 (−120.16, 109.59)	36.42 (−26.33, 99.16)
CVD				
No	(reference)	44.06 (27.31, 60.83) ***	15.79 (−5.53, 37.11)	52.39 (35.18, 69.60) ***
Yes	(reference)	59.50 (−153.63, 272.65)	219.79 (−163.06, 602.63)	115.91 (−91.75, 323.58)
**hs-CRP**				
Unadjusted	(reference)	0.10 (0.04, 0.15) **	0.08 (−0.0001, 0.15)	0.16 (0.11, 0.22) ***
Model1 †	(reference)	0.09 (0.03, 0.14) **	0.08 (−0.0003, 0.15)	0.16 (0.10, 0.22) ***
Model2 ‡	(reference)	0.07 (0.01, 0.13) **	0.07 (−0.005, 0.15)	0.14 (0.08, 0.20) ***
Subgroup effects				
Age, years ‡				
<55	(reference)	0.01 (−0.05, 0.08)	0.11 (0.04, 0.18) **	0.15 (0.09, 0.21) ***
≥55	(reference)	0.15 (0.02, 0.29) *	−0.03 (−0.21, 0.16)	0.15 (0.004, 0.30) *
Sex ‡				
Female	(reference)	0.04 (−0.05, 0.13)	0.21 (0.09, 0.33) **	0.11 (0.0003, 0.,22) *
Male	(reference)	0.08 (0.005, 0.16) *	0.03 (−0.06, 0.13)	0.14 (0.07, 0.22) ***
Diabetes ‡				
No	(reference)	0.07 (0.006, 0.13) *	0.06 (−0.02, 0.13)	0.15 (0.09, 0.22) ***
Yes	(reference)	0.12 (−0.06, 0.29)	0.94 (0.48, 1.41) ***	0.11 (−0.05, 0.27)
Hypertension ‡				
No	(reference)	0.06 (−0.01, 0.13)	0.07 (−0.005, 0.15)	0.15 (0.08, 0.22) ***
Yes	(reference)	0.07 (−0.08, 0.21)	0.08 (−0.20, 0.36)	0.11 (−0.04, 0.26)
CVD				
No	(reference)	0.06 (0.002, 0.12) *	0.08 (0.003, 0.15) *	0.14 (0.08, 0.20) ***
Yes	(reference)	0.18 (−0.27, 0.62)	−0.02 (−0.73, 0.69)	0.19 (−0.22, 0.59)

*n* = number, β = regression coefficient, CI = confidence interval, MHNO = metabolically healthy non-obesity group, MUNO = metabolically unhealthy non-obesity group, MHO = metabolically healthy obesity group, MUO = metabolically unhealthy obesity group, DM = diabetes, HTN = hypertension, CACS = coronary artery calcification score, PCF = pericardial fat, TAT = thoracic peri-aortic adipose tissue. † Model 1 is adjusted for sex and age. ‡ Model 2 is adjusted for the Framingham score. * *p* < 0.05; ** *p* < 0.01; *** *p* < 0.001.

**Table 3:** Associations between obesity, visceral adiposity, MU, and CAC burden (according to the CACS).

	Unadjusted		Model 1 †		Model 2 ‡		Interaction Coefficient	Interaction *p* Value
	β (95% CI)	*p* Value	β (95% CI)	*p* Value	β (95% CI)	*p* Value		
CACS								
All patients (*n* = 2846)								
Obesity	39.87 (23.91, 55.83) ***	<0.001	34.79 (19.23, 50.35) ***	<0.001	27.28 (11.25, 43.32) ***	0.001	1.316	0.941
MU	54.53 (40.01, 69.06) ***	<0.001	35.39 (20.95, 49.83) ***	<0.001	32.30 (16.53, 48.08) ***	<0.001		
Age < 55 (*n* = 2035)								
Obesity	25.21 (13.12, 37.31) ***	<0.001	22.68 (10.48, 34.89) ***	<0.001	19.14 (6.84, 31.45) **	0.002	20.815	0.129
MU	30.78 (19.29, 42.26) ***	<0.001	28.34 (16.73, 39.96) ***	<0.001	22.13 (9.81, 34.45) ***	<0.001		
Age ≥ 55 (*n* = 811)								
Obesity	73.39 (27.82, 118.96) **	0.002	70.03 (24.57, 115.50) **	0.003	63.12 (16.80, 109.44) **	0.008	−52.715	0.306
MU	74.87 (35.74, 114.00) ***	<0.001	74.68 (35.72, 113.64) ***	<0.001	62.35 (16.96, 107.73) **	0.007		
Female (*n* = 786)								
Obesity	54.49 (31.30, 77.67) ***	<0.001	39.49 (16.98, 62.00) ***	<0.001	41.49 (17.95, 65.04) **	0.001	−41.096	0.097
MU	49.94 (30.35, 69.53) ***	<0.001	22.72 (2.55, 42.88) *	0.027	30.02 (7.68, 52.36) **	0.009		
Male (*n* = 2060)								
Obesity	32.87 (13.03, 52.71) ***	<0.001	35.42 (16.18, 54.66) ***	0.001	20.24 (0.52, 39.95) *	0.044	10.978	0.618
MU	53.42 (35.03, 71.81) ***	<0.001	40.76 (22.72, 58.81) ***	<0.001	23.98 (4.14, 43.81) *	0.018		
High PCF	31.75 (18.39, 45.11) ***	<0.001	5.42 (−8.26, 19.09)	0.44	13.81 (−0.08, 27.71)	0.051	38.02	0.014
MU	54.53 (40.01, 69.06) ***	<0.001	35.39 (20.95, 49.83) ***	<0.001	32.30 (16.53, 48.08) ***	<0.001		
Age <55 (*n* = 2035)								
High PCF	21.03 (10.71, 31.35) ***	<0.001	14.43 (3.76, 25.10) **	0.008	14.37 (3.69, 25.05) **	0.008	16.62	0.17
MU	30.78 (19.29, 42.26) ***	<0.001	28.34 (16.73, 39.96) ***	<0.001	22.13 (9.81, 34.45) ***	<0.001		
Age ≥55 (*n* = 811)								
High PCF	14.60 (−24.45, 53.66)	0.46	−16.69 (−55.29, 21.90)	0.40	3.70 (−36.00, 43.39)	0.86	43.02	0.34
MU	74.87 (35.74, 114.00) ***	<0.001	74.68 (35.72, 113.64) ***	<0.001	62.35 (16.96, 107.73) **	0.007		
Female (*n* = 786)								
High PCF	36.69 (18.45, 54.92) ***	<0.001	7.54 (−11.42, 26.50)	0.44	20.99 (1.67, 40.31) *	0.033	36.86	0.08
MU	49.94 (30.35, 69.53) ***	<0.001	22.72 (2.55, 42.88) *	0.027	30.02 (7.68, 52.36) **	0.009		
Male (*n* = 2060)								
High PCF	25.69 (8.47, 42.90) **	0.003	5.94 (−11.16, 23.03)	0.50	5.34 (−12.23, 22.91)	0.55	34.83	0.077
MU	53.42 (35.03, 71.81) ***	<0.001	40.76 (22.72, 58.81) ***	<0.001	23.98 (4.14, 43.81) *	0.018		
High TAT	47.00 (33.63, 60.37) ***	<0.001	16.78 (1.91, 31.64) *	0.027	28.25 (14.04, 42.45) ***	<0.001	46.74	0.003
MU	54.53 (40.01, 69.06) ***	<0.001	35.39 (20.95, 49.83) ***	<0.001	32.30 (16.53, 48.08) ***	<0.001		
Age <55 (*n* = 2035)								
High TAT	31.87 (21.51, 42.22) ***	<0.001	24.68 (13.27, 36.09) ***	<0.001	24.97 (13.96, 35.97) ***	<0.001	38.30	0.002
MU	30.78 (19.29, 42.26) ***	<0.001	28.34 (16.73, 39.96) ***	<0.001	22.13 (9.81, 34.45) ***	<0.001		
Age ≥ 55 (*n* = 811)								
High TAT	44.90 (6.33, 83.47) *	0.023	−6.68 (−50.17, 36.81)	0.76	34.04 (−5.47, 73.55)	0.09	35.33	0.43
MU	74.87 (35.74, 114.00) ***	<0.001	74.68 (35.72, 113.64) ***	<0.001	62.35 (16.96, 107.73) **	0.007		
Female (*n* = 786)								
High TAT	34.63 (7.77, 61.50) *	0.012	−3.71 (−30.89, 23.47)	0.79	9.67 (−18.57, 37.91)	0.50	−18.51	0.53
MU	49.94 (30.35, 69.53) ***	<0.001	22.72 (2.55, 42.88) *	0.027	30.02 (7.68, 52.36) **	0.009		
Male (*n* = 2060)								
High TAT	46.34 (29.15, 63.53) ***	<0.001	18.46 (0.86, 36.06) *	0.04	20.37 (2.10, 38.64) *	0.029	66.69	0.002
MU	53.42 (35.03, 71.81) ***	<0.001	40.76 (22.72, 58.81) ***	<0.001	23.98 (4.14, 43.81) *	0.018		

*n* = numbers, β = regression coefficient, CI = confidence interval, BMI = body mass index, obesity was defined as BMI ≥ 27 kg/m^2^, MU=metabolic unhealthy, a combination of MUNO and MUO, MUNO = metabolically unhealthy non-obesity group, MUO=metabolically unhealthy obesity group, CACS = coronary artery calcification score, PCF = pericardial fat, TAT = thoracic peri-aortic adipose tissue. † Model 1 is adjusted for sex and age. ‡ Model 2 is adjusted for the Framingham score. * *p* < 0.05; ** *p* < 0.01; *** *p* < 0.001.

## Data Availability

No additional data are available.

## Supplementary Materials

The following are available online at https://www.mdpi.com/2075-4418/11/1/40/s1, Table S1. Associations between four obesity phenotypes, CAC burden (according to the CACS) and visceral adiposity measures, Supplemental Table S2. Associations between four obesity phenotypes, prevalent coronary calcification and subgroups analysis, Table S3. Associations between obesity and metabolic unhealthy status with CAC burden (according to the CACS), Figure S1. Prevalence coronary artery calcification (CAC) with metabolic score (MS) in non-obesity and obesity populations (Supplemental Figure S1A); and in lower and higher PCF/TAT groups (Supplemental Figure S1B).

## Abbreviations

CAC	coronary artery calcium
CACS	coronary artery calcium score
PCF	pericardial adipose tissue
TAT	Thoracic peri-aortic adipose tissue
BW	bodyweight
DM	diabetes mellitus
CAD	Coronary artery disease
MDCT	Multidetector computed tomography
CVD	Cardiovascular disease
FRS	Framingham risk score
MHO	Metabolically healthy obesity
MU	Metabolically unhealthy
MUNO	Metabolically unhealthy non-obesity
BMI	Body mass index
MHNO	Metabolically healthy non-obesity
MUO	Metabolically unhealthy obesity
WC	Waist circumstance
HC	Hip circumference
BP	Blood pressure
hs-CRP	high-sensitivity C-reactive protein
SD	standarddeviation
ANOVA	Analysis of variance
OR	Odds ratio
CI	Confidence interval
MetS	Metabolic syndrome.
