# Supplementary material for: Visceral Adiposity, Pro-Inflammatory Signaling and Vasculopathy in Metabolically Unhealthy Non-Obesity Phenotype
Source: Diagnostics (Basel). 2020 Dec 29;11(1):40. doi: 10.3390/diagnostics11010040 (PMC7824214; doi:10.3390/diagnostics11010040)
Supplement: Supplementary file 1 [file diagnostics-11-00040-s001.pdf]

Supplemental Tables

Supplemental Table S1. Associations between four obesity phenotypes, CAC burden (according to the CACS) and visceral adiposity measures.

	MHNO (n=1793)	MUNO (n=423)	MHO (n=227)	MUO (n=403)
	**β (95% CI)**	**β (95% CI)**	**β (95% CI)**	**β (95% CI)**
		**PCF**		
Unadjusted	(reference)	18.17 (15.22, 21.12)***	23.40 (19.55, 27.24)***	35.21 (32.21, 38.22)***
Model1†	(reference)	12.93 (10.12, 15.74)***	22.68 (19.08, 26.28)***	31.06 (28.21, 33.91)***
Model2‡	(reference)	9.18 (6.16, 12.19)***	21.24 (17.55, 24.92)***	27.56 (24.54, 30.57)***
Subgroup effects				
Age, years‡				
<55	(reference)	8.09 (4.63, 11.55)***	19.64 (15.81, 23.46)***	23.68 (20.37, 26.98)***
≥55	(reference)	14.44 (8.37, 20.51)***	28.57 (19.60, 37.54)***	39.89 (33.35, 46.44)***
Sex‡				
Female	(reference)	11.49 (5.68, 17.29)***	22.38 (14.97, 29.78)***	29.63 (22.43, 36.82)***
Male	(reference)	8.04 (4.52, 11.55)***	20.46 (16.26, 24.66)***	25.50 (22.16, 28.84)***
Diabetes‡				
No	(reference)	9.68 (6.57, 12.80)***	21.29 (17.66, 24.92)***	26.30 (23.17, 29.43)***
Yes	(reference)	11.28 (-2.89, 25.47)	-0.91 (-49.24, 47.43)	39.79 (25.87, 53.70)***
Hypertension‡				
No	(reference)	8.40 (4.98, 11.82)***	21.28 (17.52, 25.04)***	24.84 (21.32, 28.35)***
Yes	(reference)	12.37 (5.09, 19.65)***	21.13 (8.22, 34.03)***	33.72 (26.67, 40.77)***
CVD				
No	(reference)	2.70 (2.32, 3.07)***	2.77 (2.30, 3.25)***	5.24 (4.86, 5.62)***
Yes	(reference)	2.40 (0.30, 4.50)*	6.61 (2.83, 10.39)**	6.91 (4.86, 8.96)***
		**TAT**		
Unadjusted	(reference)	2.67 (2.31, 3.04)***	2.89 (2.42, 3.37)***	5.42 (5.04, 5.79)***
Model 1†	(reference)	1.87 (1.56, 2.17)***	2.63 (2.23, 3.02)***	4.55 (4.23, 4.86)***
Model 2‡	(reference)	1.44 (1.07, 1.81)***	2.60 (2.14, 3.05)***	4.36 (3.99, 4.73)***
Subgroup effects				
Age, years‡				
<55	(reference)	1.55 (1.13, 1.96)***	2.53 (2.08, 2.99)***	3.72 (3.33, 4.12)***
≥55	(reference)	1.78 (1.01, 2.55)***	2.97 (1.83, 4.11)***	6.18 (5.35, 7.01)***
Sex‡				
Female	(reference)	1.15 (0.74, 1.56)***	2.08 (1.56, 2.61)***	3.27 (2.76, 3.78)***
Male	(reference)	1.42 (0.99, 1.85)***	2.58 (2.07, 3.09)***	4.02 (3.61, 4.42)***
Diabetes‡				
No	(reference)	1.38 (1.00, 1.76)***	2.63 (2.19, 3.07)***	4.13 (3.75, 4.51)***
Yes	(reference)	2.55 (0.71, 4.38)**	-2.39 (-8.65, 3.88)	6.36 (4.56, 8.17)***
Hypertension‡				
No	(reference)	1.37 (0.97, 1.76)***	2.58 (2.15, 3.02)***	3.63 (3.22, 4.03)***
Yes	(reference)	1.47 (0.45, 2.48)**	2.55 (0.74, 4.36)**	5.37 (4.39, 6.36)***
CVD				
No	(reference)	17.51 (14.49, 20.53)***	22.50 (18.66, 26.34)***	33.79 (30.69, 36.89)***
Yes	(reference)	22.26 (6.59, 37.93)**	50.64 (22.49, 78.79)**	44.89 (29.61, 60.16)***

Supplemental Table S2. Associations between four obesity phenotypes, prevalent coronary calcification and subgroups analysis.

	MHNO (n=1793)	MUNO (n=423)	MHO (n=227)	MUO (n=403)
	**OR (95% CI)**	**OR (95% CI)**	**OR (95% CI)**	**OR (95% CI)**
Unadjusted	1.00	2.58 (2.07, 3.21)***	1.21 (0.89, 1.64)	2.64 (2.11, 3.30)***
Model1†	1.00	1.81 (1.42, 2.31)***	1.23 (0.87, 1.72)	2.19 (1.71, 2.81)***
Model2‡	1.00	1.58 (1.24, 2.01)***	1.19 (0.87, 1.63)	1.81 (1.42, 2.30)***
Subgroup effects				
Age, years‡				
20-54	1.00	1.77 (1.28, 2.44)***	1.32 (0.90, 1.95)	2.14 (1.59, 2.89)***
≥55	1.00	1.37 (0.94, 2.01)	1.10 (0.61, 1.97)	1.70 (1.11, 2.62)*
Sex‡				
Female	1.00	2.93 (1.73, 4.96)***	1.94 (0.95, 3.98)	3.72 (2.00, 6.92)***
Male	1.00	1.36 (1.04, 1.79)*	1.00 (0.70, 1.43)	1.48 (1.14, 1.92)**
Diabetes ‡				
No	1.00	1.62 (1.25, 2.09)***	1.22 (0.89, 1.68)	1.61 (1.25, 2.09)***
Yes	1.00	-	-	-
Hypertension‡				
No	1.00	1.51 (1.13, 2.02)**	1.21 (0.86, 1.71)	1.49 (1.11, 2.02)**
Yes	1.00	1.04 (0.64, 1.68)	0.92 (0.38, 2.19)	1.39 (0.87, 2.23)
CVD‡				
No	1.00	1.65 (1.29, 2.12)***	1.16 (0.84, 1.61)	1.78 (1.38, 2.28)***
Yes	1.00	0.53 (0.19, 1.48)	1.99 (0.37, 10.72)	1.11 (0.42, 2.96)

n=number, OR=odds ratio, CI=confidence interval, MHNO=metabolically healthy non-obesity group, MUNO=metabolically unhealthy non-obesity, MHO=metabolically healthy obesity group, MUO=metabolically unhealthy obesity group, CACS=coronary artery calcification score, DM=diabetes, HTN=hypertension, CVD=cardiovascular disease.

†Model 1 is adjusted for sex and age.

‡Model 2 is adjusted for the Framingham score.

-: One group with HTN had 100% CAS.

*p<0.05; **p<0.01; ***p<0.001.

Supplemental Table S3. Associations between obesity and metabolic unhealthy status with CAC burden (according to the CACS).

	Unadjusted		Model 1		Model 2		Interaction coefficient	Interaction P value
	ORs (95% CI)	P value	OR (95% CI)	P value	OR (95% CI)	P value		
All patients (n=2846)								
Obesity	1.65 (1.37, 1.98)***	<0.001	1.58 (1.29, 1.95)***	<0.001	1.40 (1.15, 1.70)***	<0.001	-0.036	0.869
MU	2.55 (2.15, 3.02)***	<0.001	1.94 (1.61, 2.34)***	<0.001	1.65 (1.37, 2.00)***	<0.001		
Age 20-54 (n=2035)								
Obesity	1.78 (1.40, 2.25)***	<0.001	1.54 (1.21, 1.97)***	<0.001	1.5 (1.25, 2.03)***	<0.001	-0.085	0.758
MU	2.47 (1.97, 3.09)***	<0.001	2.16 (1.71, 2.71)***	<0.001	1.89 (1.49, 2.41)***	<0.001		
Age ≥55 (n=811)								
Obesity	1.59 (1.13, 2.23)**	0.007	1.53 (1.09, 2.16)*	0.015	1.34 (0.94, 1.90)	0.102	0.122	0.751
MU	2.03 (1.52, 2.72)***	<0.001	2.09 (1.55, 2.82)***	<0.001	1.49 (1.08, 2.04)*	0.014		
Female (n=786)								
Obesity	2.64 (1.70, 4.09)***	<0.001	1.90 (1.13, 3.21)*	0.016	2.02 (1.26, 3.25)**	0.004	-0.424	0.394
MU	4.82 (3.28, 7.07)***	<0.001	2.33 (1.50, 3.63)***	<0.001	2.97 (1.89, 4.68)***	<0.001		
Male (n=2060)								
Obesity	1.35 (1.10, 1.66)**	0.004	1.50 (1.20, 1.88)***	<0.001	1.21 (0.98, 1.50)	0.082	0.085	0.730
MU	2.05 (1.69, 2.48)***	<0.001	1.82 (1.48, 2.23)***	<0.001	1.43 (1.16, 1.76)***	0.001		
With DM (n=163)								
Obesity	1.9 7 (1.01, 3.83)*	0.047	2.05 (1.00, 4.21)	0.051	2.12 (1.07, 4.21)*	0.031	21.600	0.999
MU	1.99 (0.98, 4.02)	0.056	1.90 (0.88, 4.10)	0.103	1.68 (0.81, 3.50)	0.165		
Without DM (n=2683)								
Obesity	1.53 (1.26, 1.86)***	<0.001	1.50 (1.21, 1.87)***	<0.001	1.30 (1.06, 1.60)*	0.012	-0.205	0.372
MU	2.32 (1.94, 2.78)***	<0.001	1.86 (1.52, 2.27)***	<0.001	1.58 (1.29, 1.92)***	<0.001		
With HTN (n=479)								
Obesity	1.24 (0.85, 1.80)	0.263	1.37 (0.91, 2.05)	0.127	1.28 (0.87, 1.90)	0.215	0.380	0.455
MU	1.50 (1.04, 2.18)*	0.031	1.40 (0.94, 2.08)	0.099	1.22 (0.83, 1.81)	0.313		
Without HTN (n=2367)								
Obesity	1.46 (1.16, 1.82)**	0.001	1.46 (1.14, 1.87)**	0.003	1.26 (1.00, 1.59)	0.054	-0.200	0.434
MU	2.16 (1.76, 2.65)***	<0.001	1.81 (1.44, 2.27)***	<0.001	1.47 (1.17, 1.84)***	<0.001		
With CVD (n=125)								
Obesity	1.74 (0.84, 3.61)	0.138	1.46 (0.61, 3.49)	0.395	1.65 (0.76, 3.62)	0.208	0.051	0.959
MU	1.22 (0.59, 2.51)	0.595	0.98 (0.40, 2.43)	0.971	0.72 (0.31, 1.64)	0.432		
Without CVD (n=2721)								
Obesity	1.58 (1.31, 1.92)***	<0.001	1.57 (1.27, 1.95)***	<0.001	1.35 (1.10, 1.66)**	0.004	-0.076	0.739

MU	2.54 (2.13, 3.04)***	<0.001	2.02 (1.66, 2.45)***	<0.001	1.68 (1.39, 2.05)***	<0.001

n=number, OR=odds ratio, CI=confidence interval, BMI=body mass index, obesity was defined as BMI ≥27 kg/m^2, MU= metabolic unhealthy=combination of MUNO and MUO, MUNO=metabolically unhealthy non-obesity group, MUO=metabolically unhealthy obesity group, DM=diabetes, HTN=hypertension, CVD=cardiovascular disease.

†Mode l 1 is adjusted for sex and age.

‡ Model 2 is adjusted for the Framingham score.

*p<0.05; **p<0.01; ***p<0.001.

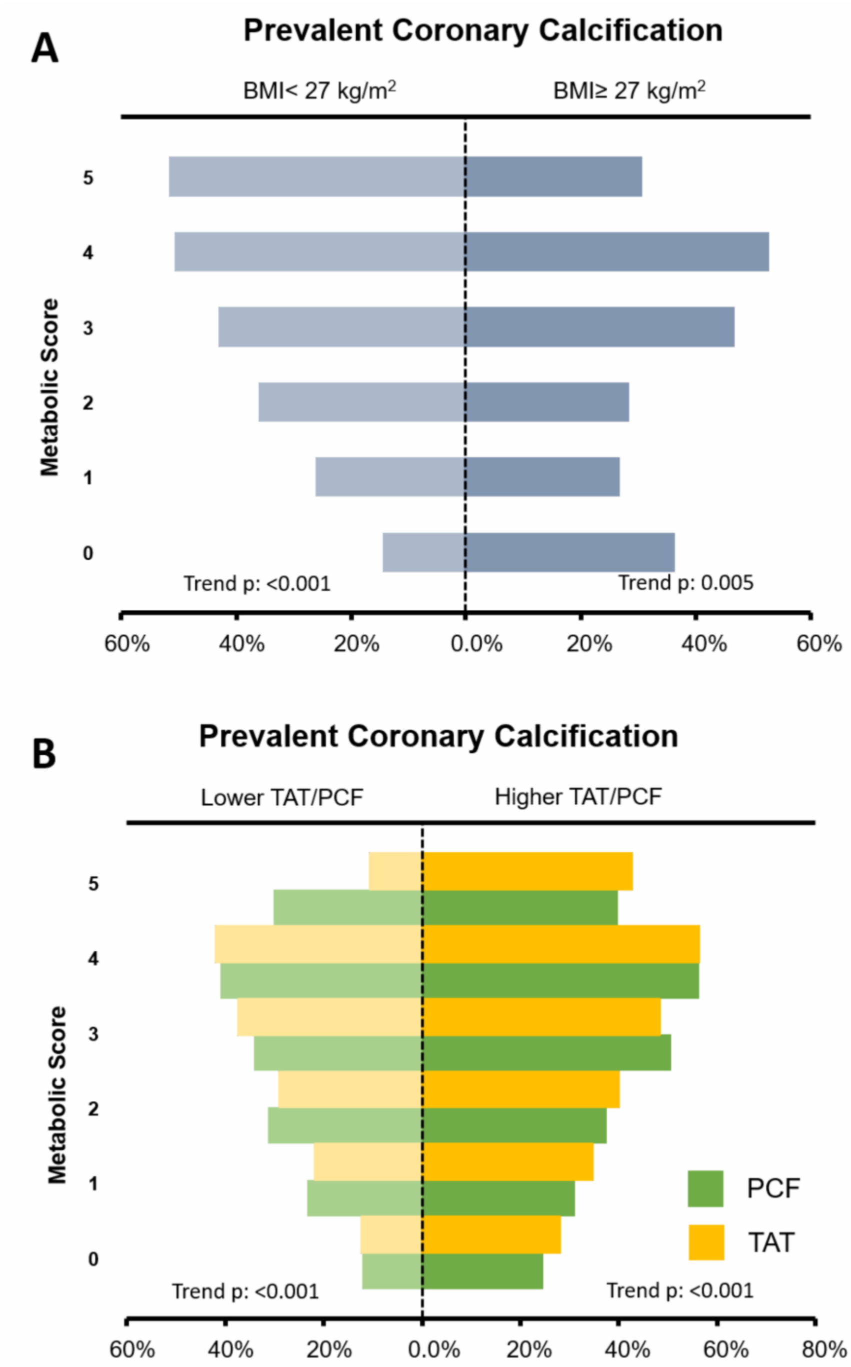

Supplemental Figure S1. Prevalence coronary artery calcification (CAC) with metabolic score (MS) in non-obesity and obesity populations (Supplemental Figure S1A); and in lower and higher PCF/TAT groups (Supplemental Figure S1B).
